# Role of Sarcopenia in Predicting 1-Year Outcomes After Mini-invasive Surgical or Endovascular Repair of Infrarenal Abdominal Aortic Aneurysms

**DOI:** 10.1093/ejcts/ezaf241

**Published:** 2025-07-18

**Authors:** Pasqualino Sirignano, David Barillà, Giulia Colonna, Chiara Pranteda, Arianna Pignataro, Carmen Emanuela Setteducati, Stefano Brizzi, Noemi Baronetto, Maurizio Taurino, Efrem Civilini

**Affiliations:** Vascular and Endovascular Surgery Unit, Sant’Andrea Hospital of Rome, Department of General and Specialistic Surgery, “Sapienza” University of Rome, Rome, 00135/00139, Italy; Vascular and Endovascular Surgery Unit of Humanitas Clinical and Research Center—IRCCS, Rozzano Department of Biomedical Sciences, Humanitas University, Milan, 20089, Italy; Vascular and Endovascular Surgery Unit, Sant’Andrea Hospital of Rome, Department of Molecular and Clinical Medicine, “Sapienza” University of Rome, 00189 Rome, 00189, Italy; Vascular and Endovascular Surgery Unit, Sant’Andrea Hospital of Rome, Department of Molecular and Clinical Medicine, “Sapienza” University of Rome, 00189 Rome, 00189, Italy; Vascular and Endovascular Surgery Unit of Humanitas Clinical and Research Center—IRCCS, Rozzano Department of Biomedical Sciences, Humanitas University, Milan, 20089, Italy; Vascular and Endovascular Surgery Unit, Sant’Andrea Hospital of Rome, Department of Molecular and Clinical Medicine, “Sapienza” University of Rome, 00189 Rome, 00189, Italy; Vascular and Endovascular Surgery Unit of Humanitas Clinical and Research Center—IRCCS, Rozzano Department of Biomedical Sciences, Humanitas University, Milan, 20089, Italy; Vascular and Endovascular Surgery Unit of Humanitas Clinical and Research Center—IRCCS, Rozzano Department of Biomedical Sciences, Humanitas University, Milan, 20089, Italy; Vascular and Endovascular Surgery Unit, Sant’Andrea Hospital of Rome, Department of Molecular and Clinical Medicine, “Sapienza” University of Rome, 00189 Rome, 00189, Italy; Vascular and Endovascular Surgery Unit of Humanitas Clinical and Research Center—IRCCS, Rozzano Department of Biomedical Sciences, Humanitas University, Milan, 20089, Italy

**Keywords:** abdominal aortic aneurysm, endovascular aneurysm repair, open repair, enhanced recovery, frail patients, sarcopenia, psoas area

## Abstract

**Objective:**

To evaluate the outcome of frail patients electively treated for abdominal aortic aneurysm (AAA) by open surgery with enhanced repair protocol (OSER) or endovascular aneurysm repair (EVAR).

**Design:**

A retrospective study on frail AAA patients treated by EVAR and OSER was conducted. Patients were defined as frail if they present a normalized total psoas muscle area (nTPA) <500 mm^2^/m^2^. This study aimed to evaluate the association between sarcopenia and AAA-related as well as all-cause mortality rates. Secondary outcomes included reinterventions, operative time, blood transfusion, length of intensive care unit (ICU), and postoperative hospital stay.

**Results:**

A total of 403 patients were included in the study, of which 122 (30.3%) had a nTPA < 500mm^2^/m^2^. Among them, 272 (67.5%) patients were treated with EVAR while 131 (32.5%) with OSER. Although EVAR was more frequently performed in sarcopenic patients than OSER (*P*<0.001), there were no significant differences between the 2 groups in terms of intraoperative and postoperative outcomes. Likewise, no statistically significant differences were found regarding mortality and reintervention rates at Kaplan-Meier analysis. However, sarcopenic patients undergoing OSER exhibited a significantly higher all-cause mortality rate at 1 month (*P* = 0.031) and cumulative follow-up (*P* = 0.004) compared to all other subgroups.

**Conclusions:**

The present experience demonstrates that less invasive approaches, but surgical or endovascular, are viable for AAA patients with no significant difference in intraoperative and immediate postoperative outcomes. Nevertheless, the potential of EVAR as a preferred strategy should be considered for frail patients based on ascertained sarcopenia.

## Graphical Abstract

**Figure:**
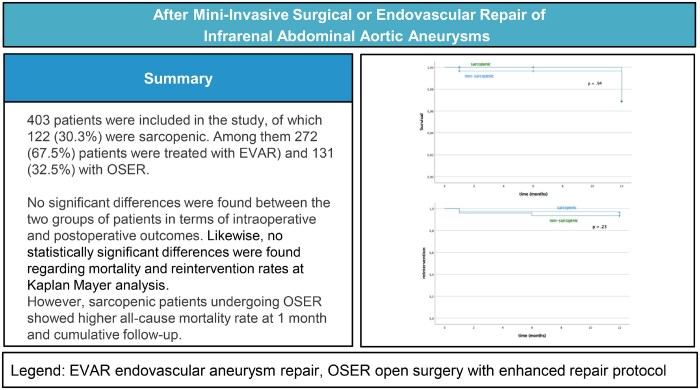

## INTRODUCTION

Elderly patients with abdominal aortic aneurysms (AAA), particularly those with severe comorbidities, are often considered unsuitable for surgical or endovascular treatment.^1^ A thorough preoperative assessment, combined with prognostic stratification and risk scores measuring patient’s frailty status, is crucial for careful patient selection.^2^

Several parameters have been proposed to define frailty. Among these, sarcopenia has been associated with various diseases and adverse health outcomes including diminished quality of life and increased overall mortality.^3^ In patients with aortic aneurysm, sarcopenia has demonstrated a negative prognostic impact by increasing the operative risk and serving as a potential predictor of all-cause mortality.^4^^,^^5^ Sarcopenia, defined as the loss of subcutaneous fat and muscle hypotrophy associated with fat accumulation, can be identified using computed tomography angiography (CTA). This is typically assessed by measuring the psoas muscle area (PMA) at the level of the third or fourth lumbar vertebra (L3 or L4).^6^

Minimally invasive treatment options have been proposed in frail patients. Endovascular aneurysm repair (EVAR) is considered the first-line option in elderly, with better short-term outcomes.^7^^,^^8^ Open surgery with enhanced recovery (OSER) protocols have been shown to reduce surgical stress and accelerate postoperative recovery with perioperative burdens comparable to EVAR.^9–11^ Only few studies dealing with the results of these 2 minimally invasive procedures in sarcopenic patients have been reported. Moreover, the prognostic value of sarcopenia in predicting outcomes after elective aortic repair remains controversial, with studies reporting conflicting results.

The present study was conducted retrospectively to assess how frailty, defined through the lens of sarcopenia, affects postoperative and long-term outcomes in patients electively treated with aortic surgery. Furthermore, the study aims to discern the variations in these outcomes based on the choice of surgical approach (EVAR vs OSER).

## METHODS

The present study enrolled all patients treated between March 2015 and December 2021 for infrarenal AAA. Indication for AAA repair was based primarily on aneurysm diameter, rate of growth more than 1 cm/years, and aortic wall morphology were also considered, as advised from the EACTS/STS aortic guidelines.^12^ All standard endovascular (EVAR) procedures performed at Vascular and Endovascular Surgery Unit of Sant’Andrea Hospital (Rome) and all enhanced recovery (OSER) protocols performed at Endovascular Surgery Unit of Humanitas Clinical and Research Center—IRCCS (Rozzano) were included.

Exclusion criteria included cases of EVAR performed outside the device’s instruction for use (IFU), chimney EVAR or fenestrated device implantations, OR without ER, and patients lacking at least 1 postoperative computed tomographic angiography (CTA). Urgent cases were also excluded from the study.

Patients were classified as “frail” if they exhibited sarcopenia as determined by CTA, following the criteria set forth by Cheng and colleagues.^13^ Specifically, sarcopenia was defined by a normalized total psoas muscle area (nTPA) less than 500 mm^2^/m^2^. Psoas muscle cross-sectional area was measured from CTA images acquired within 60 days before the procedure, with measurements taken at the midpoint of the third lumbar vertebra by a trained user. Calculation and normalization of data to obtain a standardized nTPA were facilitated using a Microsoft Excel formula:


$$\mathrm{nTPA}:\;\left(\mathrm{Right}\;\mathrm{Psoas}\;\mathrm{Area}\;in\;mm^{2}+\mathrm{Left}\;\mathrm{Psoas}\;\mathrm{Area}\;in\;mm^{2}\right)/\mathrm{height}\;in\;m^{2}.$$

During preoperative evaluation, a standardized assessment of all comorbidities was conducted at both centres through clinical history, physical examination, chest radiography, electrocardiography, pulmonary function testing, transthoracic echocardiography, and laboratory testing.^14^

### EVAR protocol

Patients underwent CTA of the entire thoracic and abdominal aorta to evaluate the presence of other aortic lesions and to determine the feasibility of EVAR. All measurements were performed using a workstation with dedicated software (Horos v4.0.0 RC open-source software; https://horosproject.org/#content-1).^15^ EVAR procedures were all performed with commercially available devices according to their IFU; endograft selection, type of anaesthesia, surgical or percutaneous access, and necessity for adjunctive intraoperative procedures and type of anaesthesia, were tailored for each patient.^16^

### OSER protocol

Preoperatively, the OSER protocol includes a meeting with a trained nurse at least 3 weeks before surgery to address risk factor modification and to educate the patient about the perioperative period. On the day of the procedure, antihypertensive medications (sartans, calcium channel blockers, and ACE inhibitors) are discontinued to improve intraoperative haemodynamic control. Patients are prescribed pregabalin (50-75 mg) starting the night before surgery and continuing until discharge.^17^ Additionally, a carbohydrate load is administered the evening before and the morning of surgery, including 800 mL of a 12.5% maltodextrin-containing clear drink the night before and 400 mL up to 2 hours before anaesthesia.

During the intraoperative phase, the protocol starts with administering 2 g of cefazolin for antibiotic prophylaxis. In line with standard enhanced recovery protocols, central venous catheters (CVCs) were no longer routinely placed, Instead, minimally invasive haemodynamic monitoring via arterial access (radial artery cannulation) is preferred. While CVCs and arterial lines provide different types of information, dynamic parameters derived from arterial waveform analysis, such as stroke volume variation (SVV), pulse pressure variation (PPV), cardiac output (CO), and stroke volume index (SVI), offer more clinically relevant insights into fluid responsiveness and intravascular volume status compared to static measurements like central venous pressure (CVP).^18^

Body temperature is maintained using forced-air warming devices and warmed intravenous fluids. Fluid administration is closely monitored and controlled based on haemodynamic parameters. Before making the incision, analgesia is performed by subcutaneous infiltration of lidocaine and ropivacaine along the incision line. To minimize tissue handling, evisceration is avoided by using self-retaining retractors to ensure optimal surgical access. At the end of the procedure, no drains are placed, but a perifascial analgesic catheter is inserted beneath the rectus abdominis sheath. Additionally, the nasogastric tube is removed at the conclusion of surgery.

Postoperatively, patients are awakened immediately following surgery, with admission to the intensive care unit (ICU) reserved only for high-risk cases. For the first 24 hours, patients are monitored in the ward using multi-parameter systems. Opioids, prokinetics, and intravenous hydration are avoided immediately after surgery. Postoperative nausea and vomiting are managed with intravenous ondansetron (4-8 mg). Oral hydration is initiated 4 hours after returning to the ward, and patients are allowed a modest dinner the evening of surgery. Early mobilization begins 4 hours from ward admission, supported by an abdominal band, with active mobilization continuing from the first postoperative day. On the same day, the urinary catheter and multi-parameter monitors are removed. By the second postoperative day, the perifascial analgesic catheter is removed, and an enema is administered if bowel discharge has not occurred. Patients are typically discharged on the second or third postoperative day.^18^

### Follow-up protocol

After EVAR, the follow-up protocol included physical examination, duplex-ultrasound scan (DUS), and CT at 30 days. DUS was then performed at 3 and 6 months, at 1 year. All patients underwent CT imaging 1 year after the initial procedure, without further CT examinations in the absence of complications detected by DUS follow-up.

After the OSER procedure, the patient is evaluated after 2 weeks at the outpatient clinic where physical evaluation is performed, and stitches are removed. Thereafter, a DUS and vascular surgeon evaluation is performed at 3 months, 6 months, and 1 year.

### Study outcomes

The primary objective of this study was to evaluate the association between sarcopenia and all-cause mortality rates as well as reinterventions. Secondary outcomes included the need for reinterventions, operative time, blood transfusion requirements, length of postoperative hospital stay, ICU admission, and duration of ICU stay. Additionally, outcomes of sarcopenic and non-sarcopenic patients were evaluated separately, based on the type of treatment received (EVAR or OSER).

### Statistical analysis

Demographic and clinical data were displayed as mean (±standard deviation) for normally distributed data, as median (25th to 75th percentile) for non-parametric data, and as frequencies (percentages) for the categorical data.

The distribution of continuous variables was assessed using the Kolmogorov-Smirnov test. All continuous variables (age, nTPA, AAA diameter, procedural time, ICU stay, hospital stay) resulted to be normally distributed and were compared using the Independent sample T-test. Categorical variables (sex, hypertension, diabetes, dyslipidaemia, coronary artery disease, stroke/TIA, chronic kidney disease, COPD, smoking history, obesity, American Society of Anesthesiologists [ASA] score, iliac involvement, type of repair, anaesthesia, adjunctive procedures, blood transfusion, need for ICU stay, and postoperative outcomes), were compared using the Chi-square test or Fisher’s exact test, depending on cell counts. Survival and time free from reintervention rates were calculated and presented using Kaplan-Meier curves and compared between 2 groups using the Log-Rank test.

A 2-tailed *P* value <0.05 was considered statistically significant. All statistical analyses were performed using Statistical Package for the Social Sciences (SPSS) version 27.0 (IBM Corp, Armonk, NY, USA, 2020).

The study adhered to the principles outlined in the Declaration of Helsinki,^19^ and patients provided consent for procedures and data collection and analysis. Given the retrospective nature of the study, it adhered to Italian Law by notifying local ethical committees, obviating the need for formal approval. No formal sample size calculation was performed, as this was a retrospective observational study based on all eligible cases available from the 2 participating centres. Given the exploratory nature of the present analysis, the findings should be interpreted with caution.

## RESULTS

Among the 897 patients treated for AAA in both centres, 403 patients met the inclusion criteria and were enrolled during the entire study period, of whom 272 subjected to EVAR (67.5%) and 131 subjected to OSER (32.5%).

Demographic data, preoperative risk factors, and anatomical features for all enrolled patients are described in **Table 1**. One-hundred twenty-two out of 403 included patients (30.27%) had a nTPA < 500mm^2^/m^2^. Beside a lower preoperative nTPA (414 ± 67 vs676 ± 128mm^2^/m^2^; *P* = 0.010), sarcopenic patients were older (*P* < 0.001), and affected by COPD (*P* = 0.009; OR: 0.56, CI 95%:0.37-0.87). Preoperative ASA score was significantly different between the 2 groups (*P* <0.001), while no significant differences in AAA anatomical features were found (**Table 1**).

**Table 1.** Demographic, Clinical Characteristics, and Anatomical Features of All Patients Included in Present Series

	Patients	Non-sarcopenic	Sarcopenic	*P* value
	(*n* = 403)	(*n* = 281)	(*n* = 122)	(OR; CI 95%)
Age year (mean ± SD)	73.3 ± 7.5	71.9 ± 7.3	76.6 ± 6.6	< 0.001
Male sex (*n*; %)	367; 91	273; 97.1	94; 77	< 0.001
Hypertension (*n*; %)	352; 87.3	247; 87.9	105; 86.1	0.61
Diabetes (*n*; %)	66; 16.3	46; 16.4	20; 16.4	0.99
Dyslipidemia (*n*; %)	268; 66.5	191; 67.9	77; 63.1	0.34
CAD (*n*; %)	156; 38.7	106; 37.7	50; 40.9	0.53
Stroke/TIA (*n*; %)	30; 7.4	20; 7.1	10; 8.2	0.70
CKD (*n*; %)	48; 11.9	30; 10.7	18; 14.7	0.24
COPD (*n*; %)	166; 41.2	104; 37	62; 50.8	0.009
Smoking history (*n*;%)	264; 65.5	188; 66.9	76; 62.3	0.37
Obesity (*n*; %)	97; 24.1	65; 23.1	32; 26.2	0.10
ASA score				
I (*n*; %)	12; 2.9	11; 3.9	1; 0.9	<0.001
II (*n*; %)	92; 22.8	70; 24.9	22; 18	
III (*n*; %)	280; 69.4	187; 66.5	93; 76.2	
IV (*n*; %)	15; 3.7	11; 3.9	4; 3.3	
Unknown (*n*; %)	4; 1.2	2; 0.8	2; 1.6	
nTPA (mean ± SD)	597 ± 171.3	676 ± 128	414 ± 67	0.01
AAA diameter mm (mean ± SD)	55.3 ± 10.4	54.7 ± 10.1	56.6 ± 12.6	0.11
Iliac involvement (*n*; %)	109; 27	76; 27.1	33; 27.1	0.99

Abbreviations: AAA: abdominal aortic aneurysm; ASA: America Society of Anesthesiologists; CAD: coronary artery disease; CKD: chronic kidney disease; COPD: chronic obstructive pulmonary disease; N: number; nTPA: normalized total psoas areas; OR: odds ratio.

EVAR was performed in 272 patients (67.5%), while OSER in 131 patients (32.5%). More technical procedural details are shown in **Table 2**.

**Table 2.** Intra- and Postoperative Details of the Present Cohort Divided for Type of Treatment (EVAR vs OSER)

	403 Patients	272 EVAR	131 OSER
Local anaesthesia (*n*; %)	72; 17.9	72; 26.5	–
General anaesthesia (*n*; %)	331; 82.1	200; 73.5	131; 100
Aorto-aortic aneurysm (*n*; %)	93; 23.1	–	93; 70.9
Aorto-iliac aneurysm (*n*; %)	38; 9.4	–	38; 29.1
Need for adjunctive procedures (*n*; %)	23; 5.7	23; 8.4	–
Embolization (*n*; %)	15; 3.7	15; 5.5	–
Single IBD implantation (*n*; %)	8; 1.9	8; 2.9	–
Procedural time min (mean ± SD)	128.9 ± 39.8	122.6 ± 39.5	141.7 ± 38.8
Need for blood transfusion (*n*; %)	37; 9.2	24; 8.8	13; 9.9
Need for ICU stay (*n*; %)	57; 14.1	49; 18	8; 6.1
ICU stay hours (mean ± SD)	11.5 ± 4.5	10.79 ± 4.13	15.62 ± 4.37
Hospital stay days (mean ± SD)	4.77 ± 3.44	4.82 ± 2.87	4.28 ± 2.37

Abbreviations: EVAR: endovascular aneurysm repair; IBD: iliac branched device; ICU: intensive care unit; OSER: open surgery with enhanced recovery.

Details of commercially available devices implanted in the present series are reported in **Table 3**.

**Table 3.** Details of Commercially Available Endografts Implanted in Patients Who Underwent EVAR in Present Series

Implanted endograft	272 EVAR
AFX/AFX2 (*n*; %)	37; 13.6
Aorfix (*n*; %)	4; 1.5
Excluder/C3/Conformable (*n*; %)	76; 27.9
Endurant I/II/Iis (*n*; %)	73; 26.8
Nellix (*n*; %)	28; 10.3
Prime/iX/Alto (*n*; %)	42; 15.4
Treo (*n*; %)	1; 0.4
Zenith (*n*; %)	11; 4.1

Abbreviation: EVAR: endovascular aneurysm repair.

Immediate intraoperative technical success was achieved in all patients. At 30-day follow-up, 2 patients (0.49%) died following OSER due to non-AAA-related causes. Endoleaks were observed in 68 patients. Graft patency was maintained in all but 10 patients (97.5%). Reinterventions were required in 12 patients (2.97%): 2 following OSER (1 for retroperitoneal haematoma drainage and 1 for bowel resection) and 10 following EVAR (3 for endoleak corrections, 6 for limb fibrinolysis, and 1 for common femoral artery pseudoaneurysm repair). Three additional deaths were recorded during follow-up (**Table 4**).

**Table 4.** Clinical Characteristics of Patients Who Died During Follow-up

Cause of death	Time (months)	Index procedure	Sarcopenia
Oesophageal cancer	1	OSER	yes
Infection non-graft related	1	OSER	no
AMI	6	EVAR	yes
Lung cancer	6	OSER	no
Biliary sepsis	12	OSER	yes

Abbreviations: AMI: acute myocardial infarction; EVAR: endovascular aneurysm repair; OSER: open surgery with enhanced recovery.

Despite EVAR was more frequently used in sarcopenic patient compared to OSER (*P* <0.001), no differences were registered in terms of procedural time, need for blood transfusion, and postoperative ICU stay. However, 18% of sarcopenic patients required ICU admission, and 9.8% needed blood transfusions, indicating a higher morbidity trend in this group compared to non-sarcopenic patients.(**Table 5**).

**Table 5.** Intra- and Postoperative Details of the Present Cohort Divided in Sarcopenic and Non-sarcopenic Patients

	Non-sarcopenic	Sarcopenic	*P* value
	(*n* = 281)	(*n* = 122)	(OR; CI 95%)
Type of repair			
EVAR (*n*; %)	173; 61.5	99; 81.1	0.0001
OSER (*n*; %)	108; 38.5	23; 18.9	
Anesthesia			
Local (*n*; %)	44; 15.7	28; 33	0.079
General (*n*; %)	237; 84.3	94; 77	
Adjunctive procedures (*n*; %)	14; 4.9	9; 7.4	0.34
Procedural time min (mean ± SD)	130.18 ± 44.59	126.04 ± 40.33	0.347
Need for blood transfusion (*n*; %)	25; 8.9	12; 9.8	0.76
Need for ICU stay (*n*; %)	35; 12.4	22; 18	0.13

Abbreviations: EVAR: endovascular aneurysm repair; ICU: intensive care unit; N: number; OR: odds ratio; OSER: open surgery with enhanced recovery.

**Figure 1.**
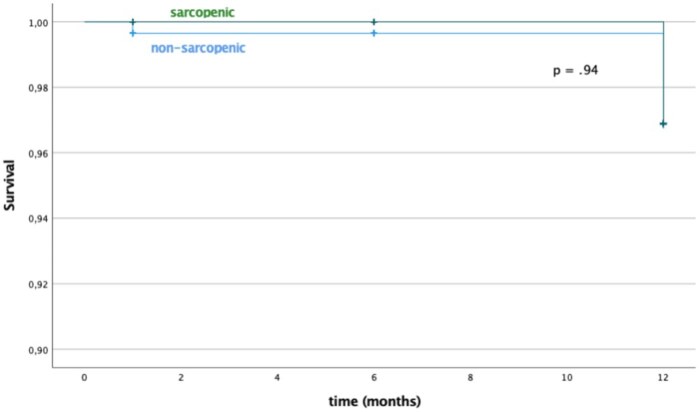
Survival Rates of Sarcopenic and Non-sarcopenic Patients

**Figure 2.**
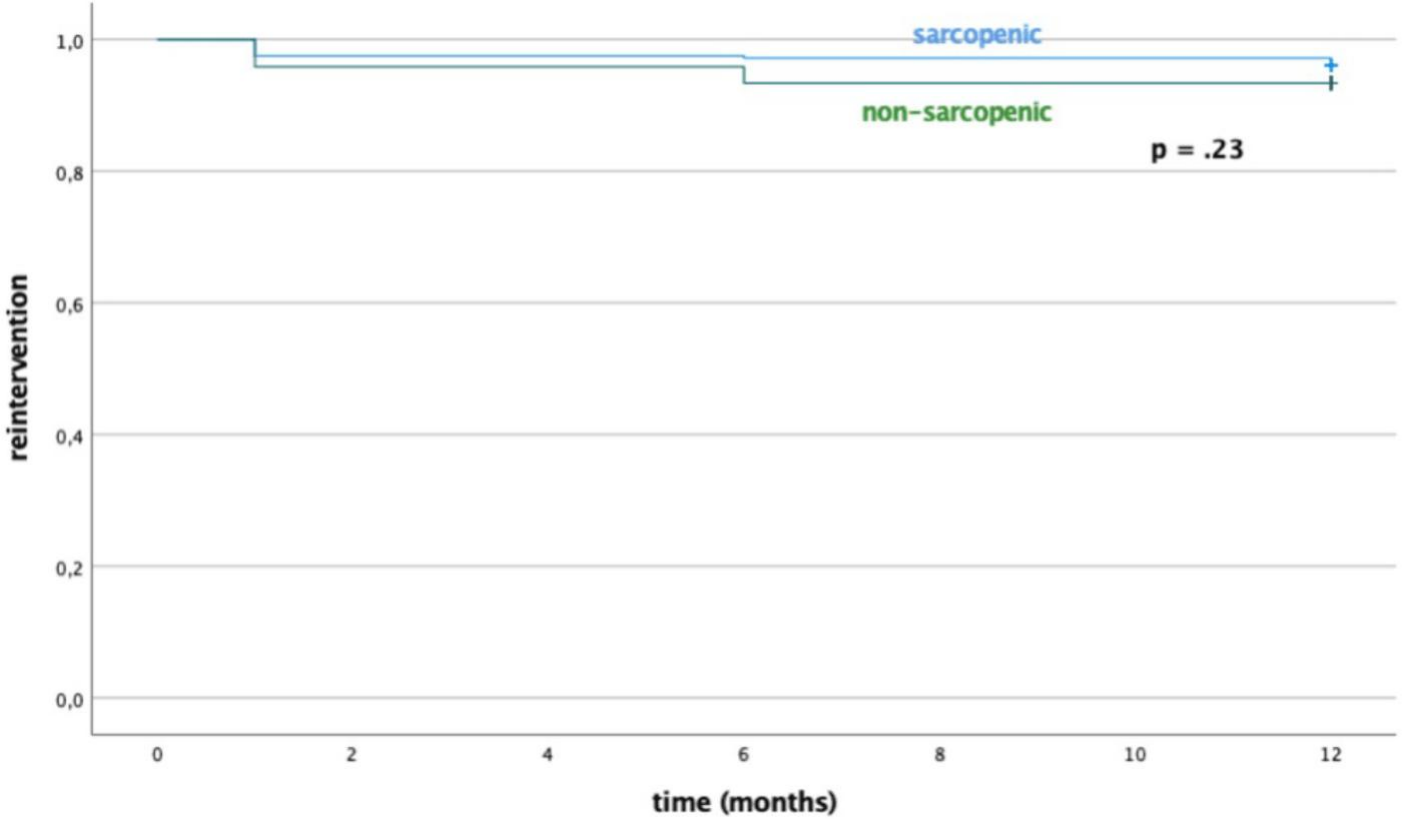
Reintervention Rates of Sarcopenic and Non-sarcopenic Patients

At Kaplan-Meier analysis, estimated survival rates at 1, 6, and 12 months were, respectively, 99.5%, 99.5%, and 96.5% for sarcopenic patients, and 99.5%, 99.5%, and 98.8% for non-sarcopenic patients (**Figure 1**). Estimated time free from reintervention at 1, 6, and 12 months were, respectively, 99%, 94%, and 92% for sarcopenic patients and 99.5%, 96%, and 94% for non-sarcopenic patients (**Figure 2**). No statistical difference was found in terms of survival (*P* = 0.92) and reintervention (*P* = 0.23) rates between sarcopenic and non-sarcopenic patients.

When considering the survival rates divided for the type of treatment (EVAR vs OSER), sarcopenic patients undergoing OSER demonstrated a significantly higher all-cause mortality rate, both at 1 month (*P* = 0.031) and during overall follow-up (*P* = 0.004), compared to all other sub-groups.

## DISCUSSION

The present study demonstrates that intraoperative and immediate postoperative outcomes were comparable between sarcopenic and non-sarcopenic patients subjected to OSER and EVAR. However, sarcopenic patients treated with OSER showed significantly poorer survival rates in comparison to those treated by EVAR.

Current European and Italian guidelines recommend treatment for aortic abdominal aneurysm (AAA) with a diameter ≥ 5.5 cm.^20^^,^^21^ However, since this cutoff may not be sensitive enough for elderly patients with low life expectancy, additional parameters besides aortic diameter must be considered.

Sarcopenia has emerged as a useful and easily accessible marker to stratify patients undergoing aortic repair.^22^ This is the first study analysing outcomes in sarcopenic patients treated with EVAR and OSER.

The demographic characteristics of our cohort indicate a higher prevalence of sarcopenia among females compared to males. This finding is consistent with previous studies^23^^,^^24^ and may represent an additional factor to consider when analysing sex-related differences in outcomes following elective aortic repair. However, this association remains controversial,^25^ warranting further investigation.

The present research reveals that EVAR was more frequently performed than OSER in sarcopenic patients. Given the better short-term benefits of EVAR compared to open treatment,^26^ it is largely recognized as the first-line option for elderly. Sarcopenia is known to be age-related, without any other cause,^27^ and consistent results were obtained in the present study.

No statistically significant differences were found between sarcopenic and non-sarcopenic patients treated with EVAR and OSER in terms of intraoperative, postoperative outcomes (**Tables 2 and 5**), reintervention and survival rates (**Figures 1 and 2**). These results are consistent with earlier studies.^22^^,^^28^^,^^29^

Interestingly, when mortality rates are calculated separately for EVAR and OSER subgroups, sarcopenic patients treated with OSER showed significantly worse outcomes compared to the rest of the cohort. Recent studies have challenged the assumption that elderly and frail patients necessarily experience better outcomes with EVAR compared to open repair, suggesting that all-cause mortality is not significantly influenced by the type of surgical approach.^1^ However, these findings impose of a careful selection of frail individuals, potentially fit for open surgery, even with an enhanced recovery protocol.

The role of sarcopenia is still controversial in current literature.^28–31^This study contributes to the ongoing discussion by providing additional data that confirm sarcopenia alone should not be considered a contraindication for elective aortic surgery.

The present study shows some limitations. First, being a retrospective study, it carries an inherent selection bias and limits the ability to account for potential confounding variables. The absence of prospective data and standardized protocols across institutions further constrains the applicability of these results. Secondly, data were collected from 2 different centres, with their distinct criteria for patient selection. Other factors, beyond sarcopenia, may have potentially influenced the outcomes of the study, and “hospital-driven” bias may be present. Third, the definition of sarcopenia as nTPA < 500mm^2^/m^2^ is not the only one reported in literature. Therefore, the application of different thresholds or parameters could have potentially altered the results of the present study. Lastly, potential confounders such as age and COPD may have influenced survival outcomes, thereby limiting the strength of the conclusions drawn.

In conclusion, our findings suggest that the 2 different surgical approaches do not significantly affect intraoperative and immediate postoperative outcomes, supporting the feasibility of less invasive approaches. Additional parameters are required to improve clinical stratification. Concerning mortality in frail patients, even in a small series, EVAR demonstrated better survival rates than OSER, highlighting the potential of EVAR as a preferred strategy for sarcopenic patients. Nevertheless, these results need to be taken with caution and further research are needed to validate them.

## Data Availability

The data underlying this article will be shared on reasonable request to the corresponding author.
